# Palaeoenvironmental evolution of formation of Bayanjargalan oil shale: evidence from trace elements and biomarkers

**DOI:** 10.1038/s41598-021-83415-6

**Published:** 2021-02-25

**Authors:** Youhong Sun, Demchig Tsolmon, Xuanlong Shan, Wentong He, Wei Guo

**Affiliations:** 1 Construction Engineering College, Jilin University, ChangChun, 130000 People’s Republic of China; 2 National-Local Joint Engineering Laboratory of In-Situ Conversion, Drilling and Exploitation Technology for Oil Shale, Changchun, 130021 Jilin People’s Republic of China; 3 Key Laboratory of Drilling and Exploitation Technology in Complex Condition, Ministry of Land and Resource, Changchun, 130026 Jilin People’s Republic of China; 4 College of Earth Sciences, Jilin University, ChangChun, 130000 People’s Republic of China

**Keywords:** Biogeochemistry, Environmental sciences, Solid Earth sciences

## Abstract

The genetic type of the Bayanerhet Formation oil shale in the Bayanjargalan mine area is an inland lacustrine oil shale deposit. Inorganic element analysis and organic geochemical testing of oil shale samples collected in three boreholes show that the Bayanerhet Formation oil shale has relatively high organic contents, e.g., average TOC values of 6.53, 7.32 and 8.84 (corresponding to oil contents of 5.49%, 6.07% and 7.50%) in boreholes BJ3807, BJ3405 and BJ3005, respectively. Analysis of organic matter sources with biomarkers indicates that lower aquatic organisms such as algae contribute more to the organic matter than higher plants do. According to research on the values of Fe2O3/FeO, Rb/Sr and w (La) n/w (Yb)n in cores from the three boreholes, the Bayanjargalan oil shale is inferred to have formed in a humid paleoclimate with a relatively high sedimentation rate. In research on the evolution of the paleoaquifer in which the oil shale formed, the values of Fe^3+^/Fe^2+^, V/V + Ni, Ni/V, Ceanom and δCe are applied as sensitive indicators of the redox conditions in the aqueous medium. These values indicate that the Bayanjargalan oil shale formed in a water body with a weak redox environment. Moreover, the values of Ca/(Ca + Fe) and Sr/Ba and the values of gammacerane/αβC30 hopane in biomarkers show that the oil shale was formed in a saltwater environment. Analysis of Mo and U shows high endogenous lake productivity, corresponding to high TOC, which suggests that the lacustrine productivity played an important role in organic matter enrichment. The Lower Cretaceous Bayanerhet Formation (K1bt) in the Bayanjargalan mine area encompasses a complete sequence and was formed during lowstand, transgression, highstand and regression periods. The dominant oil shale deposits were formed in the transgression system tract and high stand system tract, and these oil shales have a high oil content and stable occurrence. A large set of thick, high-TOC and high-oil-content oil shales in the second member of the Bayanerhet Formation was deposited under such conditions. The abundant terrigenous supply under warm and humid conditions significantly promoted the primitive biological productivity, and the weak redox saltwater environment had relatively high productivity. All the favorable conditions promoted the formation of high-quality oil shale.

## Introduction

The Bayanjargalan Basin is a secondary small-scale half-graben basin in the northeastern Niyalga Basin in Mongolia. Abundant oil shale developed in the Lower Cretaceous Bayanerhet Formation in this basin. Few research data on the Bayanerhet Formation oil shale is available. Previous research on the sedimentary geology of oil shale in the Bayanjargalan Basin has determined that the Bayanerhet Formation (K1bt) was deposited during the Early Cretaceous faulting period in the Bayanjargalan Basin^1^. The oil shale formed during the highstand systems tract (HST), is both sapropelic and sapropelic-humic, and belongs to deep lacustrine deposits^1^. In this paper, inorganic element composition and analysis of organic matter in organic-rich samples are combined with geological characteristics to reconstruct the paleogeographic conditions under which the oil shale formed.

## Geological setting

### Tectonic evolution and sequence stratigraphy of the Bayanjargalan basin

**Figure 1:**
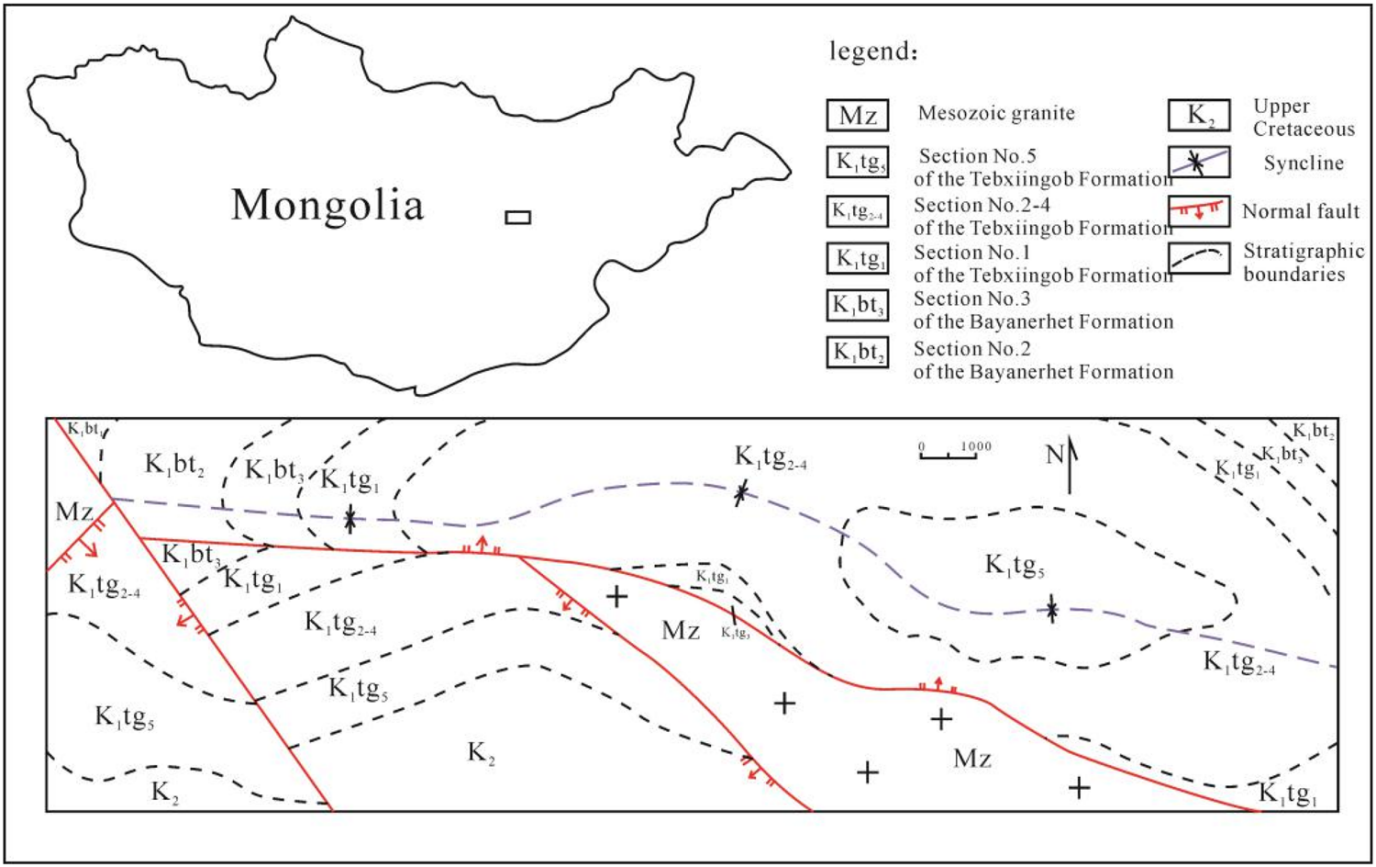
Geological map of the Bayanjargalan Basin.

The Bayanjargalan Basin is located in the middle section of fold belt in northern Mongolia. Affected by Late Jurassic-Early Cretaceous faulting in north central Mongolia, a series of secondary graben and half-graben basins formed inside the Bayanjargalan Basin, which is composed of small-scale faulted lake basin groups in terms of geologic characteristics. These basins were developed on the basement of the Hercynian fold belt. Each lake basin has its independent sedimentary system, sedimentary center, and marginal facies Fig. 1. The basin generally has an E–W trend and well-developed secondary E–W and S–N-trending structures. The secondary small-scale half-graben petroliferous basin has a nearly E–W trend. Locally affected by faulting, the formation has steeper dip, which is less than 20°. This region is flat bottom land and partially covered. The deeply weathered strata are dominantly covered by Quaternary formations, and the bedrock is only locally exposed in hills. Late Jurassic (J_3_), Cretaceous (K), and Quaternary Holocene (Qh) strata are developed from bottom to top.

### Bayanerhet Formation

**Figure 2:**
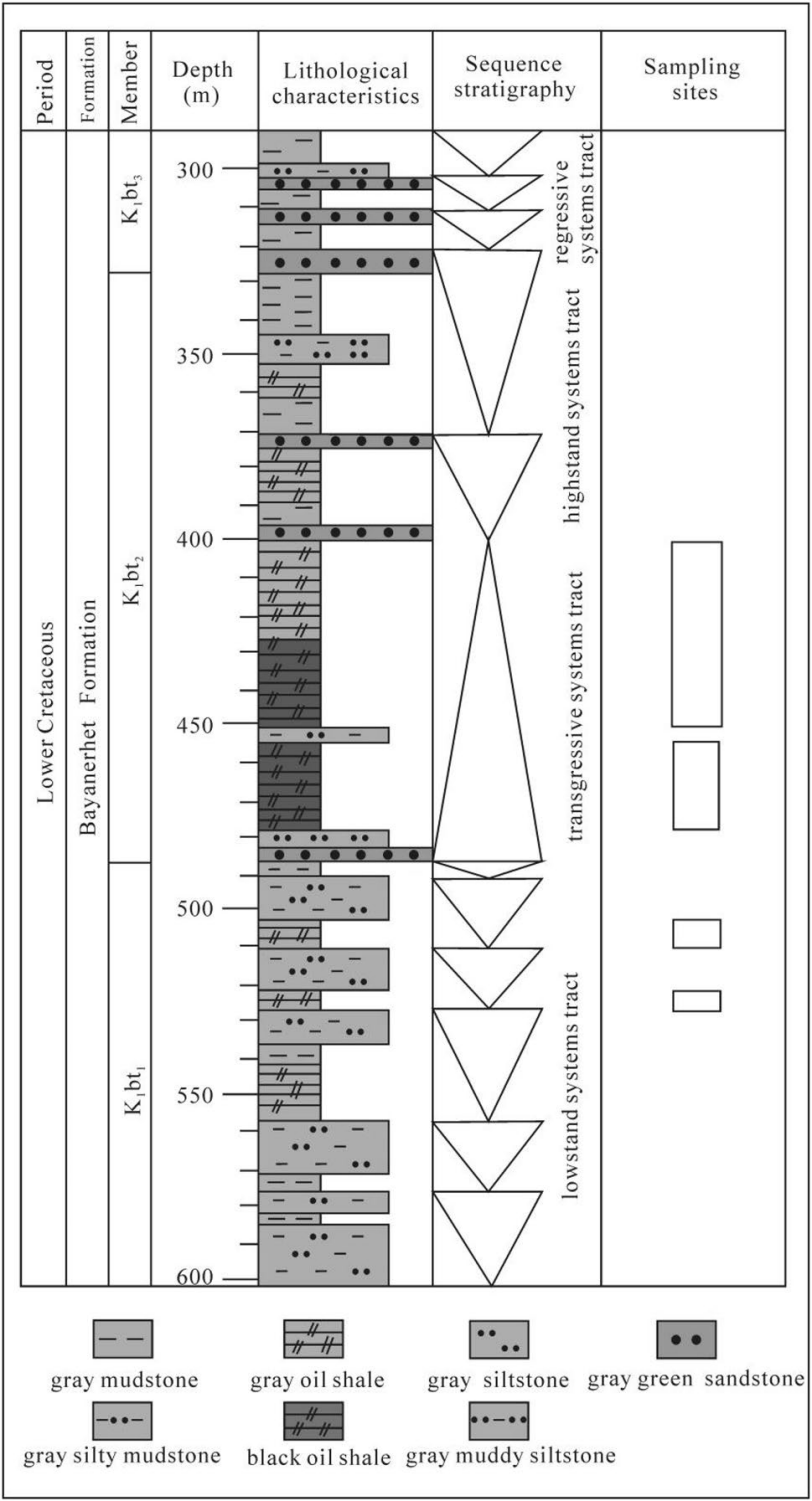
Stratigraphic subdivision of the Bayanjargalan Basin.

The Bayanerhet Formation is composed of deposits from the early basin depression period, which had a wide sedimentary range. The lower part is dominated by deep and semideep lacustrine mudstone and shale intercalated with oil shale. The Bayanerhet Formation has an average thickness of 280 m and a burial depth of oil shale between 450 and 500 m, and the formation is subdivided into the K_1_bt_1_, K_1_bt_2_, and K_1_bt_3_ members (Fig. 2)^1^.

### (1) The first member of the Bayanerhet Formation (K_1_bt_1_)

The lithology of K_1_bt_1_ is dominated by gray-green and gray calcareous siltstone, calciferous siltstone, sandstone, and pebbled sandstone. The upper interval is locally intercalated with thin layers of oil shale and shale. K_1_bt_1_ has gastropods, ostracods, Phyllopoda, and algae. The formation has an average thickness of 45 m and is in unconformable contact with the underlying strata.

### (2) The second member of the Bayanerhet Formation (K_1_bt_2_)

K_1_bt_2_ contains the oil-bearing strata and is distributed in the western and eastern parts of the study area. This member is dominated by oil shale, shale, mudstone, and siltstone and has zoolites such as gastropods, ostracods, Phyllopoda, and fish. The formation thickness is between 26.15 m and 124.75 m, averaging 82.54 m.

### (3) The third member of the Bayanerhet Formation (K_1_bt_3_)

K_1_bt_3_ is distributed in the western and eastern parts of the study area and is dominated by dark gray mudstone, silty mudstone, and shale. The lower formation has thin layers of oil shale occasionally seen, pure mudstone, fine texture, and horizontal bedding. K_1_bt_3_ has zoolites such as gastropods, ostracods, and fish. The formation thickness is between 66.35 m and 159.60 m, averaging 105.04 m.

## Materials and methods

### Materials

The study area covers the southern part of the central depression and the western and eastern parts of the southeast uplift. Samples were collected from the oil shale in the Bayanjargalan mine area, which formed in the Mesozoic Lower Cretaceous Bayanerhet Formation. The samples for analysis of total organic carbon (TOC), major elements, and minor elements are from the oil shale cores encountered by boreholes BJ3807, BJ3405, and BJ3005 and are numbered with well number + serial number. Among the samples for organic geochemical tests, BJ-1 was cut from the oil shale samples in the surface outcrop, and BJ-2, BJ-3, and BJ-4 were cut from cores of borehole BJ3005.

The rocks above and below the oil shale strata are dominated by siltstone and mudstone. The oil shale is mainly dark gray, black gray, and taupe and shows jagging and conchoidal fractures. It feels oily if it is scratched and becomes rolled if it is peeled with a knife. It is ignitable with white smoke and a strong bitumen smell when it meets with open fire. The oil shale has horizontal bedding and laminae and occurs as laminar sheets, with medium- to thick-bedded and massive structures seen. It has abundant Conchostraca, algal fossils, and carbonized phytodetritus.

### Methods

Oil shale oil content test approximately 50 g of oil shale samples are heated in a low temperature furnace at a rate of 10 ℃/min in a dry open system, heated to 520 ℃ and kept warm at 2 h, and the product in the receiver is a mixture of oil and water, in which 80 mL of xylene is added. The oil and water products and xylene were boiled, the volume of shale oil in water was measured and the oil yield was calculated. Standard according to product SH/T0508-92.

The experiments were conducted in the China National & Regional Joint Engineering Laboratory of Oil Shale In situ Transformation and Drilling & Production Technologies and the State Key Laboratory of Petroleum Resources and Prospecting of China University of Petroleum with a strictly controlled experimental environment and instruments in good operating condition. Therefore, the experimental results are highly reliable.

The major and minor elements were measured in the China National & Regional Joint Engineering Laboratory of Oil Shale In-situ Transformation and Drilling & Production Technologies with high-resolution inductively coupled plasma mass spectrometry (HR-ICP-MS) on an Element I instrument manufactured by Finnigan MAT and with X-ray fluorescence spectrometry based on the Chinese National Standard DZ/T0223-2001.

An organic petrographic study was conducted in the Key Laboratory of Oil Shale and Coexistent Energy Minerals of Jilin Province. For the production of light sheets for the organic rock pyrolysis experiment analysis. The instrument used in the rock pyrolysis experiment analysis was a Rock Eval-6 analyser at the Center for Scientific Test of Jilin University (China). The parameters S1, S2, S3 and Tmax were measured, and the hydrogen index (HI) was calculated accordingly. In these methods, the amount of pyrolyzate released from kerogen was normalized to TOC to give the HI. The temperature of maximum hydrocarbon generation (Tmax) is defined at the maximum value of the S2-peak and serves as a maturity indicator.

The data quality of sample testing was controlled with parallel samples and national standard samples. Determination of the TOC and oil contents and the extraction and separation of chloroform bitumen “A” were implemented according to the Determination of Bitumen from Rocks by Chloroform Extraction (SY/T5118-2005) and Analysis of Family Composition of Rock Extract and Crude Oil by TLC–FID (SY/6T338-1997) under a temperature of 15 °C and a relative humidity of 10%. The extracted saturated hydrocarbons were analyzed with gas chromatography (GC) and gas chromatography-mass spectrometry (GC–MS). After the oil shale samples were cleaned and crushed, the organic matter in the samples was extracted in chloroform with a Soxhlet extractor, and the asphaltene in the extract was precipitated with petroleum ether and separated by column chromatography to obtain the saturated hydrocarbon components, which were analyzed by GC–MS. The analyses were conducted in the Geochemistry Laboratory, College of Geosciences, China University of Petroleum (Beijing), with an Agilent 6890GC/5975i MS (serial number 20062451) instrument and under an ambient temperature of 20 °C and a relative humidity of 40%. The analysis was implemented according to The Standard Test Method for Biomarker in Sediment and Crude-oil by GC–MS (GB/T 18606-2001). The GC conditions were as follows: high-purity (99.999%) He as the carrier gas, a flow rate of 1 mL/min, HP-5MS (60 m × 0.25 mm × 0.25 µm) as the chromatographic column, an inlet temperature of 300 °C, and unsplit stream sample injection. The GC temperature program was as follows: the temperature was initially held at 50 °C for 1 min, ramped to 120 °C at a rate of 20 °C/min, ramped to 310 °C at a rate of 3 °C/min, and held at 310 °C for 25 min. The MS conditions were as follows: an electron impact (EI) ion source and an ion source ionization energy of 70 eV; the data collection method was SCAN/SIM.

## Results

### Major and trace element test results

The major element contents, minor element contents, and average values related to the paleoenvironment in each layer of the Bayanerhet Formation oil shale are listed in Table 1 and are similar to those in the oil shale in North America^2^. The oil shale in the Bayanerhet Formation has relatively high contents of Al_2_O_3_ and SiO_2_, averaging 13.03% and 47.78%, respectively, and relatively high contents of Mn, Sr, Ba, Li and Zn^3^.

**Table 1:** Contents of major elements in oil shales from the Bayanjargalan basin.

Analyte symbol	Depth (m)	TOC	Oil content %	Al_2_O_3_ %	SiO_2_ %	CaO %	Fe_2_O3 %	FeO %	Ni ppm	V ppm	Cr ppm	Rb ppm	Sr ppm	Ba ppm	Cu ppm	U ppm	Mo ppm	Fe2O3/FeO	Ca /(Ca^+^ Fe)	V/Cr	V/(V^+^ Ni)	Sr/Ba	Rb/Sr
BJ3807-001	476.3	6.01	5	12.63	46.05	9.67	5.31	3.73	24.6	84.1	36.2	89.9	373	404	30	7.5	11	1.42	0.516836	2.32	0.77	0.92	0.24
BJ3807-002	479.5	4.761	3.78	13.1	47.44	7.67	6.33	4.56	25.4	91	33.6	100	395	206	30	7.4	10	1.39	0.413254	2.71	0.78	1.92	0.25
BJ3807-003	480.5	4.82	3.65	13.04	48.45	6.98	5.73	4.91	24	89	34.4	104	410	415	110	7.4	11	1.17	0.396141	2.59	0.79	0.99	0.25
BJ3807-004	483.8	5.701	4.73	13.11	48.99	7.62	5.69	3.58	23	96	38	90.9	408	411	30	7.3	11	1.59	0.451155	2.53	0.81	0.99	0.22
BJ3807-005	485.5	3.468	2.56	13.01	48.61	6.85	5.83	4.15	23.6	92	36.5	90.4	354	370	30	7.5	12	1.40	0.407011	2.52	0.80	0.96	0.26
BJ3807-006	489.25	5.179	4.46	12.61	48.46	6.26	5.7	3.56	24.5	91.6	32.8	95.1	335	436	30	7.6	10	1.60	0.403351	2.79	0.79	0.77	0.28
BJ3405-001	481.35	8.121	6.68	12.09	44.63	8.63	6.2	6.17	22.9	87	32.8	85.6	418	391	30	6.9	11	1.00	0.410952	2.65	0.79	1.07	0.20
BJ3405-002	483	7.354	6.19	12.73	47.17	6.43	5.78	4.35	24	93	34	95.3	328	388	40	7.8	12	1.33	0.388285	2.74	0.79	0.85	0.29
BJ3405-003	486.6	10.252	7.69	12.96	48.15	6	5.88	3.67	22.8	92.9	31.4	82.7	309	379	30	8	11	1.60	0.385852	2.96	0.80	0.82	0.27
BJ3405-004	488.5	9.41	7.24	11.75	45.61	6.84	6.55	4.72	25.1	81	31.5	35.1	254	323	30	8.1	14	1.39	0.377692	2.57	0.76	0.79	0.14
BJ3405-005	496	5.423	4.66	12.93	49.73	7.69	5.17	5.78	24.4	87	34.9	94.9	388	395	30	9.2	14	0.89	0.412554	2.49	0.78	0.98	0.24
BJ3405-006	499.6	9.43	8.98	13.12	48.34	8.12	6.41	5.94	24.3	92.2	35.7	97	403	363	30	7.8	11	1.08	0.396678	2.58	0.79	1.11	0.24
BJ3405-007	503.1	8.692	7.33	12.98	46.24	9.06	6.1	6.93	24.2	90.4	35.6	99.2	491	403	30	7.9	11	0.88	0.41014	2.54	0.79	1.22	0.20
BJ3405-008	508.4	12.871	10.46	13.57	47.42	8.67	6.14	3.79	24.8	94	37.7	99	469	440	30	6.7	7	1.62	0.466129	2.49	0.79	1.07	0.21
BJ3405-009	511.65	7.029	5.73	12.78	45.2	9.78	5.93	6.52	25.9	95	37.9	98.5	511	452	30	7.2	8	0.91	0.439946	2.51	0.79	1.13	0.19
BJ3405-010	518	8.875	7.88	12.07	44.68	10	5.26	3.87	24	98	38.7	92.9	543	439	30	7.8	9	1.36	0.522739	2.53	0.80	1.24	0.17
BJ3005-001	422.73	4.922	4.35	12.96	46.84	7.89	5.65	3.98	24	94	38.1	93.1	348	382	40	8.2	11	1.42	0.450342	2.47	0.80	0.91	0.27
BJ3005-002	424.8	7.273	6.09	12.42	47.95	8.06	5.81	5.83	23	85	33	101	385	394	30	7.4	10	1.00	0.409137	2.58	0.79	0.98	0.26
BJ3005-003	428.35	7.718	6.83	12.68	46.26	7.26	6.39	6.54	25.2	90	33.2	102	399	189	30	7.1	10	0.98	0.359584	2.71	0.78	2.11	0.26
BJ3005-004	431.26	6.346	5.65	12.99	47.05	6.76	5.94	6.83	38.6	87	34.9	55	309	367	30	7.7	10	0.87	0.346134	2.49	0.69	0.84	0.18
BJ3005-005	439.45	10.185	8.64	13.6	48.48	7.53	5.64	8.76	51.2	92.2	37.2	86	380	379	30	7.5	10	0.64	0.343365	2.48	0.64	1.00	0.23
BJ3005-006	442.75	5.56	3.85	13.41	48.18	6.88	6.03	4.63	23.2	93	33.5	81.9	322	362	40	7.4	12	1.30	0.392246	2.78	0.80	0.89	0.25
BJ3005-007	445	6.963	5.74	15.08	57.46	4.25	4.99	3.88	25.6	96	31.5	105	215	395	40	7.6	10	1.29	0.323933	3.05	0.79	0.54	0.49
BJ3005-008	448.2	6.007	4.44	13.76	50.95	7	5.99	3.71	25.5	97	36.7	106	392	405	40	8.5	13	1.61	0.419162	2.64	0.79	0.97	0.27
BJ3005-009	450.5	6.61	4.68	14.36	48.44	7.3	6.2	6.56	24.3	96.9	39.9	106	377	418	30	6.7	8	0.95	0.363908	2.43	0.80	0.90	0.28
BJ3005-010	453	9.065	7.65	13.32	47.03	8.21	5.79	5.25	23.5	90	36.8	95.2	457	431	30	6.5	9	1.10	0.426494	2.45	0.79	1.06	0.21
BJ3005-011	456.2	8.714	7.14	12.77	46.36	9.65	5.63	8.63	24.5	85	35.8	91.3	467	432	30	7.1	9	0.65	0.403597	2.37	0.78	1.08	0.20

The Bayanerhet Formation oil shale has total rare earth elements (∑REE) between 208.18 × 10^–6^ and 249.09 × 10^–6^, averaging 225.78 × 10^–6^, which is 1.30 times greater than those in the oil shale in North America; total light REEs (∑LREE) between 167.46 ppm and 195.85 ppm, averaging 179.75 ppm, which is 1.18 times greater than those in the oil shale in North America; and total heavy REEs (∑HREE) between 41.42 × 10^–6^ and 53.24 × 10^–6^, averaging 46.0 × 10^–6^, which is 0.39 times greater than those in the oil shale in North America. ∑LREE/∑HREE and (La/Yb)N reflect the differentiation of REEs in the analyzed samples and indirectly reflect the source of materials. Higher ∑LREE/∑HREE and (La/Yb)N values reflect greater enrichment in LREEs and greater depletions in HREEs. The oil shale samples have ∑LREE/∑HREE values between 3.70 and 4.21, averaging 3.91, and (La/Yb)N values between 9.27 and 11.19, averaging 10.27, which reflects obvious differentiation of LREEs and HREEs. The Ce anomalies (δCe) of the oil shale samples lie between 0.97 and 1.04, averaging 1.02, which reflects weak positive anomalies. Eu in the oil shale samples shows weak negative anomalies (Tables 1 and 2).

**Table 2:** Contents of major elements of oil shale in Bayanjargalan Basi*n.*

Analyte symbol	Depth (m)	La (× 10^–6^)	Ce (× 10^–6^)	Pr (× 10^–6^)	Nd (× 10^–6^)	Sm (× 10^–6^)	Eu (× 10^–6^)	Gd (× 10^–6^)	Tb (× 10^–6^)	Dy (× 10^–6^)	Ho (× 10^–6^)	Er (× 10^–6^)	Tm (× 10^–6^)	Yb (× 10^–6^)	Lu (× 10^–6^)	Y (× 10^–6^)	∑LREE (× 10^–6^)	∑HREE (× 10^–6^)	∑LREE /∑HREE	∑REE (× 10^–6^)	(La/Yb) N	Ce_anom_	δCe
BJ3807-001	476.3	39.80	85.70	9.80	38.50	7.60	1.13	5.80	0.90	4.80	1.00	2.80	0.40	2.70	0.42	29.00	182.53	47.82	3.82	230.35	9.99	−0.005	1.03
BJ3807-002	479.5	41.00	86.20	9.98	37.40	7.40	1.14	5.60	0.90	4.70	1.00	2.70	0.40	2.60	0.42	29.00	183.12	47.32	3.87	230.44	10.68	−0.01	1.01
BJ3807-003	480.5	42.90	89.60	10.40	41.00	8.10	1.15	6.10	1.00	5.30	1.00	3.00	0.43	2.80	0.45	31.00	193.15	51.08	3.78	244.23	10.38	−0.017	1.01
BJ3807−004	483.8	42.10	88.00	10.00	38.80	7.50	1.14	5.80	0.90	5.00	1.00	2.90	0.42	2.80	0.43	30.00	187.54	49.25	3.81	236.79	10.19	−0.014	1.02
BJ3807-005	485.5	40.50	86.60	9.73	37.80	7.60	1.23	5.80	0.90	5.00	1.00	2.90	0.43	2.90	0.47	28.00	183.46	47.4	3.87	230.86	9.46	−0.005	1.04
BJ3807-006	489.25	40.80	87.70	9.97	38.60	7.50	1.24	6.00	0.90	5.00	1.00	2.80	0.42	2.70	0.45	30.00	185.81	49.27	3.77	235.08	10.24	−0.004	1.04
BJ3405-001	481.35	38.70	81.40	9.31	37.00	7.10	1.06	5.10	0.80	4.30	0.80	2.40	0.35	2.30	0.37	25.00	174.57	41.42	4.21	215.99	11.4	−0.014	1.02
BJ3405-002	483	40.30	83.90	9.57	37.80	7.20	1.09	5.50	0.90	4.70	0.90	2.60	0.39	2.60	0.42	25.00	179.86	43.01	4.18	222.87	10.5	−0.017	1.02
BJ3405-003	486.6	38.60	82.10	9.34	36.70	7.00	1.09	5.30	0.90	4.50	0.90	2.60	0.38	2.50	0.42	24.00	174.83	41.5	4.21	216.33	10.46	−0.009	1.03
BJ3405-004	488.5	39.30	83.20	9.67	37.10	7.40	1.16	5.80	0.90	4.80	0.90	2.70	0.42	2.60	0.44	25.00	177.83	43.56	4.08	221.39	10.24	−0.01	1.02
BJ3405-005	496	41.50	92.90	10.50	41.60	8.10	1.25	6.60	1.00	5.60	1.10	3.00	0.45	3.00	0.49	32.00	195.85	53.24	3.68	249.09	9.37	0.009	1.06
BJ3405-006	499.6	39.30	80.10	9.83	35.70	7.10	1.06	5.80	0.90	4.70	0.90	2.60	0.40	2.70	0.42	27.00	173.09	45.42	3.81	218.51	9.86	−0.023	0.97
BJ3405-007	503.1	38.80	80.80	9.30	36.70	7.30	1.15	5.60	0.90	4.70	0.90	2.70	0.40	2.60	0.39	27.00	174.05	45.19	3.85	219.24	10.11	−0.017	1.01
BJ3405-008	508.4	36.30	75.20	8.72	34.30	6.70	1.06	5.10	0.80	4.40	0.80	2.50	0.37	2.50	0.39	27.00	162.28	43.86	3.7	206.14	9.84	−0.02	1.01
BJ3405-009	511.65	40.90	85.90	9.87	38.40	7.60	1.24	6.00	0.90	5.10	1.00	2.90	0.42	2.80	0.44	31.00	183.91	50.56	3.64	234.47	9.9	−0.013	1.02
BJ3405-010	518	38.30	82.60	9.35	36.80	7.80	1.22	5.90	0.90	5.10	1.00	2.80	0.43	2.80	0.42	28.00	176.07	47.35	3.72	223.42	9.27	−0.004	1.04
BJ3005-001	422.73	42.00	89.70	10.30	39.20	7.70	1.19	5.70	0.90	4.80	1.00	2.70	0.41	2.60	0.41	28.00	190.09	46.52	4.09	236.61	10.94	−0.005	1.03
BJ3005-002	424.8	39.80	83.60	9.65	37.70	7.40	1.07	5.30	0.90	4.70	0.90	2.60	0.39	2.50	0.42	26.00	179.22	43.71	4.1	222.93	10.78	−0.014	1.02
BJ3005-003	428.35	39.10	79.70	9.31	35.60	6.70	1.04	5.30	0.80	4.50	0.90	2.50	0.38	2.50	0.41	26.00	171.45	43.29	3.96	214.74	10.59	−0.023	0.99
BJ3005-004	431.26	42.10	87.80	10.20	39.30	7.60	1.17	5.70	0.90	4.80	1.00	2.80	0.42	2.70	0.44	28.00	188.17	46.76	4.02	234.93	10.56	−0.016	1.01
BJ3005-005	439.45	38.40	81.10	9.26	36.00	7.00	1.05	5.40	0.80	4.60	0.90	2.50	0.37	2.50	0.42	26.00	172.81	43.49	3.97	216.3	10.41	−0.011	1.02
BJ3005-006	442.75	39.50	83.80	9.54	37.00	7.30	1.10	5.50	0.90	4.80	0.90	2.70	0.40	2.60	0.42	27.00	178.24	45.22	3.94	223.46	10.29	−0.009	1.03
BJ3005-007	445	40.00	86.60	10.10	38.20	7.50	1.20	5.70	0.90	4.80	0.90	2.70	0.39	2.60	0.43	28.00	183.6	46.42	3.96	230.02	10.42	−0.001	1.03
BJ3005-008	448.2	41.20	87.00	9.96	38.90	7.60	1.14	5.80	0.90	4.80	1.00	2.80	0.41	2.70	0.44	29.00	185.8	47.85	3.88	233.65	10.34	−0.011	1.02
BJ3005-009	450.5	41.30	85.30	9.88	38.50	7.30	1.19	5.50	0.90	4.60	0.90	2.60	0.39	2.50	0.41	27.00	183.47	44.8	4.1	228.27	11.19	−0.02	1.01
BJ3005-010	453	37.30	77.20	8.93	33.90	6.70	1.07	5.10	0.80	4.40	0.90	2.60	0.38	2.50	0.40	26.00	165.1	43.08	3.83	208.18	10.11	−0.017	1.01
BJ3005-011	456.2	37.30	79.10	8.85	34.30	6.80	1.11	5.10	0.80	4.60	0.90	2.70	0.39	2.60	0.43	27.00	167.46	44.52	3.76	211.98	9.72	−0.007	1.04
Ball meteorite		0.31	0.81	0.12	0.60	0.20	0.07	0.26	0.05	0.32	0.07	0.21	0.32	0.21	0.03		182.53	47.82	3.82	230.35	9.99	−0.005	1.03

∑LREE is the content of light rare earth elements; ∑HREE is the content of heavy rare earth elements; ∑REE is the content of rare earth elements; ∑LREE/∑HREE is the ratio of the content of light rare earth elements to that of heavy rare earth elements; N represents the chondrite-normalized value; (La/Yb)N represents the ratio of La chondrite-normalized value to Yb chondrite-normalized value; δEu and δCe represent the anomalies for Eu and Ce; EuN, SmN, and GdN represent the measured chondrite-normalized values; δCe = CeN/(LaN × PrN)1/2; CeN, LaN, and PrN represent the measured chondrite-normalized values; Ceanom = lg(3CeN/(2LaN + NdN)).

### Organic test results

The oilyield, TOC, HI, and chloroform bitumen “A” contents are important indexes characterizing the abundance of organic matter. The test results for the TOC, Rock–Eval pyrolysis analyses, oil contents and chloroform bitumen “A” in 4 oil shale samples including one surface sample (BJ-1) and three core samples (BJ-2, 3 and 4) are listed in Table 3. The results show that the oil yield ranges from 3.16 to 5.15, Tmax is between 433 and 435 ℃, indicating that kerogen of oil shale belongs to immature stage, and the HI index is between 827.33 and 958.95 mg/g, with an average of 886.18 mg/g. The value of nonhydrocarbon + asphaltene/total hydrocarbon of oil sample components extracted from BJ-1 is 6.53, and those of oil sample components extracted from BJ-2, 3 and 4 are between 1.06 and 6.53, which is slightly lower than that of BJ-1. Higher relative proportions of hydrocarbons are obtained from the oil shale samples (20–38%), probably due to differences in redox conditions and/or clay-catalyzed transformation processes during early digenesis^4^ (Table 3).

**Table 3:** Organic geochemistry data of the oil shale samples in Bayanerhet Formation.

Analyte symbol	Lithology	Oil yield(%)	TOC	S1(mg/g)	S2(mg/g)	Tmax (℃)	HI (mg/g)	EOM	Asphalt wt%	Sat HC wt%	Arom wt%	NOS wt% (nonhydrocarbon )	NOS + asphalt/total hydrocarbon content
BJ-1	Oil shale	3.16	5.35	3.46	46.43	433	867.8505	0.2787	5.19	17.92	15.57	46.46	6.53
BJ-2	Oil shale	4.88	7.43	1.25	71.25	435	958.9502	0.2283	1.60	14.36	11.70	69.15	1.54
BJ-3	Oil shale	5.15	7.45	1.57	66.35	433	890.604	0.3435	1.09	34.35	21.30	35.87	1.31
BJ-4	Oil shale	4.64	6.22	1.86	51.46	433	827.3312	0.3204	4.55	22.73	28.79	50.00	1.06

**Figure 3:**
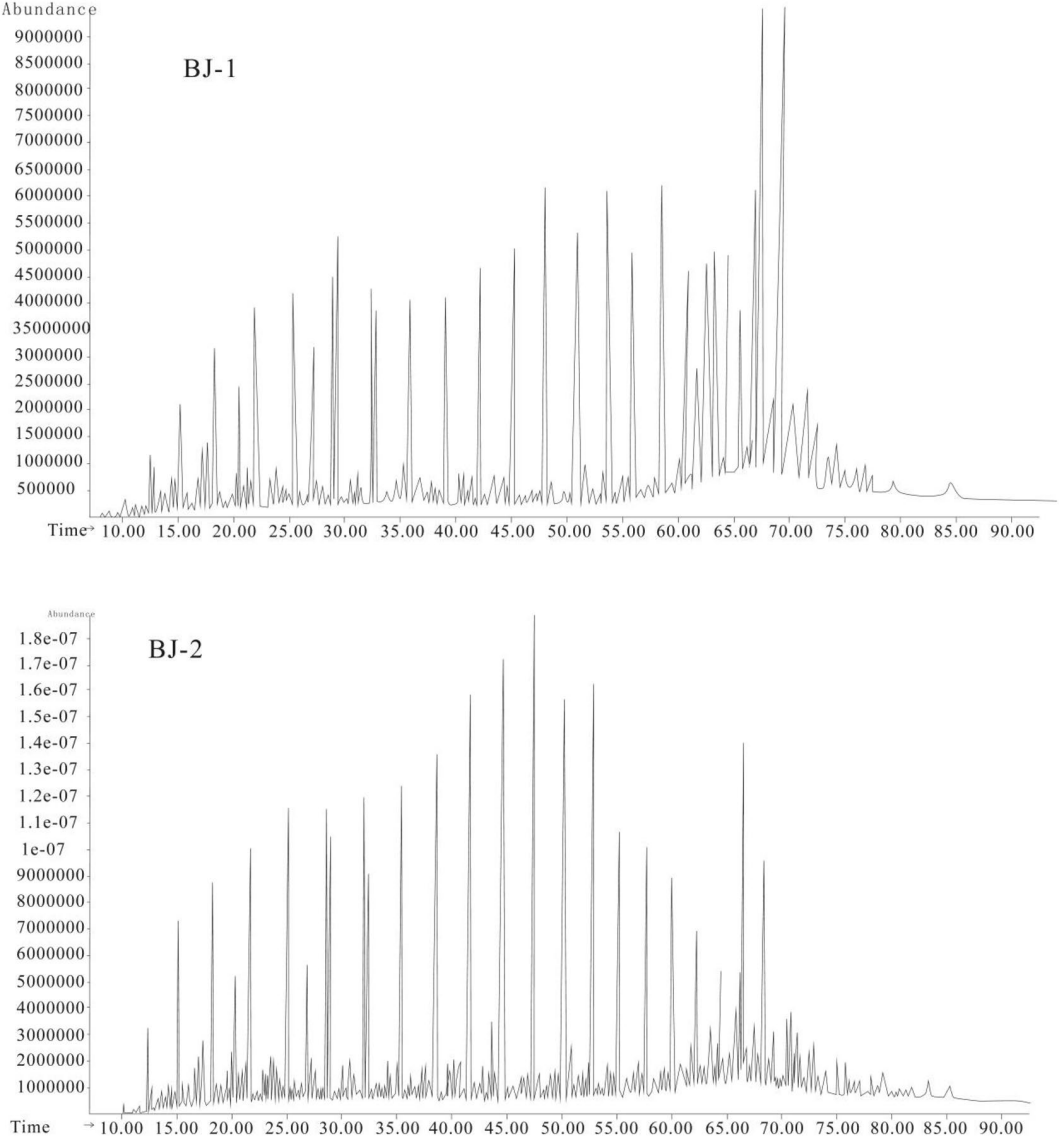
Total ion current.

The GC and GC–MS analyses of extracted saturated hydrocarbons show that the n-alkane mode of oil shale is mainly long-chain n-alkane, which shows an obvious even carbon preference (carbon preference index (CPI) between 1.28 and 1.53) The maximum abundance is between n-C27 and n-C29 according to Bray and Evans^5^ (Fig. 3). The maturity biomarkers of organic matter in source rock extracts and shale oil, such as C29 20S/(20S + 20R)(0.12 and 0.16)and C_32_ 17α(H)21(H)-hopanes(0.25 and 0.31), etc., have been studied. The results show that these biomarker parameters all show that the source rocks in the Hengtongshan Formation have low thermal maturity^6^.

The GC analyses of extracted saturated hydrocarbons show that the n-alkane mode of oil shale is mainly long-chain n-alkane, which shows an obvious even carbon preference The maximum abundance is between n-C27 and n-C29.

## Discussion

### The source of organic matter in oil shale

The genetic type of the Bayanerhet Formation oil shale in the Bayanjargalan mine area is an inland lacustrine oil shale deposit. The oil-bearing mudstone, shale, oil shale and sandstone beds generally consist of large amounts of phytodetritus, fish and lamellibranch fossils. The oil shale has abundant organic components, reflecting relatively stable lake water during the sedimentation period and favorable natural ecological conditions, which greatly benefitted biological productivity. The abundant organic matter was deposited at the bottom of the lake together with mud and silt. In the reducing environment of the deep water and the stagnant water surface, with the activities of anaerobic bacteria and the penetration of silt, sapropelic matter with abundant mineral impurities was formed, which provided the basis for the development of oil shale.

The tests of oil shale samples from the three boreholes shows average TOC values of 6.53, 7.32 and 8.84 in boreholes BJ3807, BJ3405 and BJ3005, corresponding to oil contents of 5.49%, 6.07% and 7.50%, respectively, which exceed the oil content cutoff grade of 3.5% and the commercial grade of 5% in oil shale. The oil shale has good quality and development value.

To further discuss the importance of the organic matter source for the oil shale, the biomarkers in the saturated hydrocarbon GC–MS were identified in the oil shale extract. A large portion of long-chain n-alkanes (C27–C31) typically represents the higher plants and occurs as the main component of vegetable wax^7^. Traces of short-chain alkanes in algae and microorganisms were detected in the oil shale samples.

Huang and Meischenin^8^ advanced the idea that the proportions of C27 sterane, C28 sterane, and C29 sterane relative to the corresponding homologs can be used to determine organic matter sources and their corresponding contributions (Table 4). In regular steranes, the relative contents of C27–C29 regular steranes serve as comparison indexes with high specificity to imply the organic matter sources. Algae are the main producer of C27 sterane, while C29 sterol is usually related to land plants^6^. In this study, the sources of sitosterol or stigmasterol revealing the dominance of the C29 stereoisomer is in line with the main organic matter sources of land plants^6,9^. The main bases are that the Bayanerhet Formation oil shale was formed in the Cretaceous, C27 sterane is biologically derived from lower aquatic organisms and algae, C28 sterane is biologically derived from phytoplankton mainly including diatoms, coccoliths, and dinoflagellates, and C29 sterane originates from higher plants and algae.

**Table 4:** Relative proportions of C_27_ sterane, C_28_ sterane, and C_29_ sterane.

Analyte symbol	αααC_27_ sterane/m (αααC_27_–C_28_–C_29_ sterane)	αααC_28_ sterane/m (αααC_27_–C_28_–C_29_ sterane)	αααC_29_ sterane/m (αααC_27_–C_28_–C_29_ sterane)
BJ-1	0.52	0.22	0.26
BJ-2	0.63	0.15	0.22

**Figure 4:**
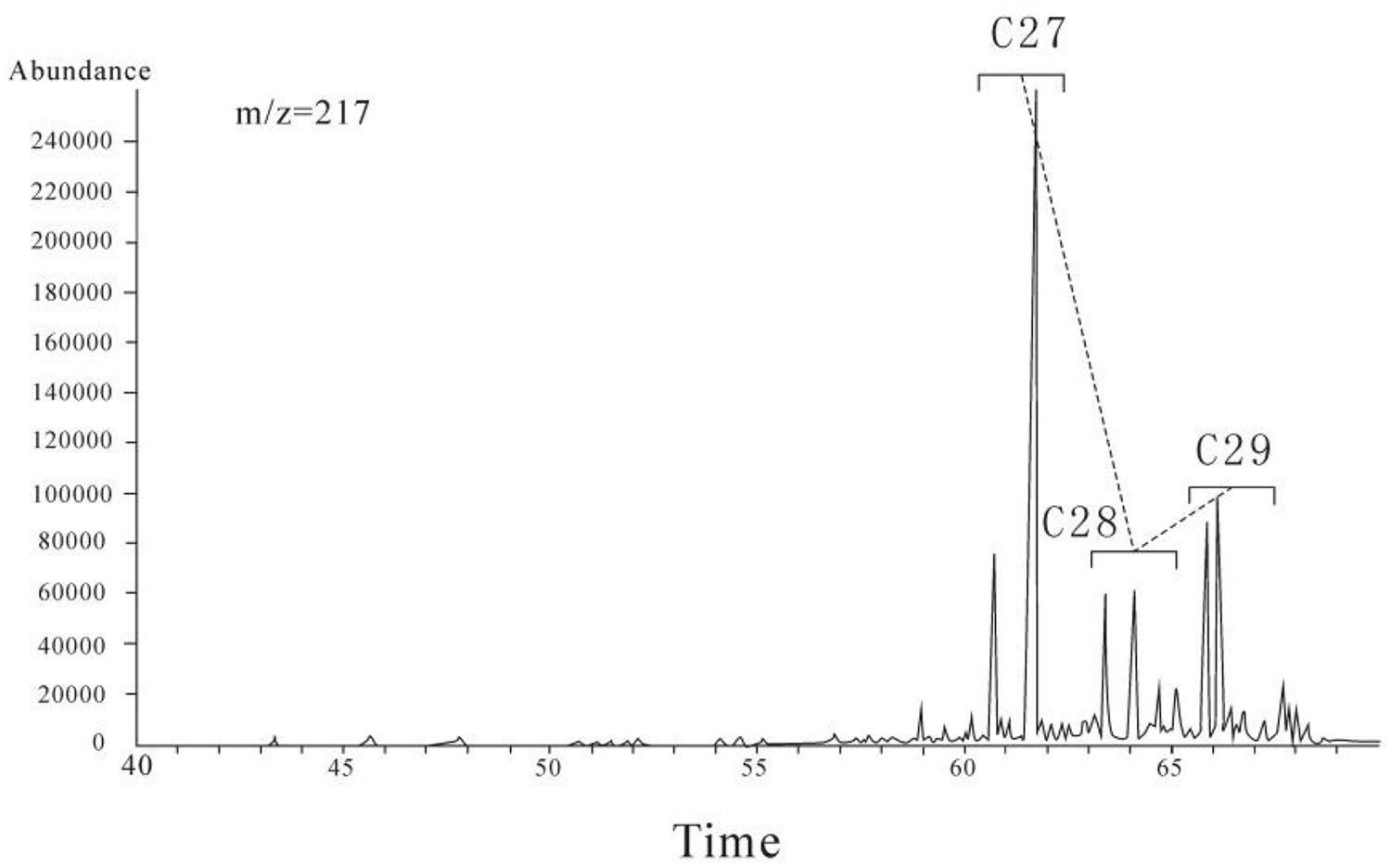
MS with m/z = 217.

In the samples, the C28 sterane abundance is relatively low, αααC27 sterane /m (αααC27-C28-C29 sterane) averages 0.57, and αααC29 sterane /m(αααC27-C28-C29 sterane) averages 0.24. Consequently, C27 >  > C28 < C29 is obtained. The C27 sterane, C28 sterane, and C29 sterane are distributed in an “L” shape in MS with m/Z = 217. This result indicates that the lower aquatic organisms such as algae contribute more to the organic matter than the higher plants do Fig. 4.

### Oil shale formation environment

The climate conditions that promote biological productivity are favorable for the formation of oil shale. Biological mass propagation and mass death cause the accumulation of a large amount of organic matter and consequently the development of high-quality oil shale.

Rb/Sr in lacustrine deposits is very sensitive to paleoclimate changes. Rb/Sr above 0.5 is generally considered to indicate an arid climate, and Rb/Sr below 0.5 a humid climate^10,11^. Rb/Sr records paleoclimate information well and sensitively reveals the medieval warm period in Daihai Lake and changes in temperature, dryness, and humidity for the Qinghai Lake area^12^. Core test analyses of the three wells show that Rb/Sr averages are 0.25, 0.21 and 0.26.Rb/Sr correlates moderately well with CaO, with a few anomalous values.Sr is relatively well correlated to CaO. This indicates the likelihood that Rb/Sr is mainly controlled by calcite content (Table 1).The Rb/Sr and CaO of the second member of the Bayanerhet Formation oil shale indicate that the oil shale was formed in a warm and humid climate.

A large set of thick, high-TOC and high oil yield oil shale in the second member in the Bayanerhet Formation was deposited under such conditions. The abundant terrigenous supply under warm and humid conditions significantly promoted the primitive biological productivity. The overlying strata were formed when the climate became semihumid and arid and the development of lake was constrained. Sediments become coarser and have more arenaceous components upward, and the lithology is dominated by a grayish-green argillaceous component and siltstone.Comprehensively analyzing paleoclimate conditions through Fe_2_O_3_/FeO and Rb/Sr is feasible.

### Analysis of the ancient water properties

**Figure 5:**
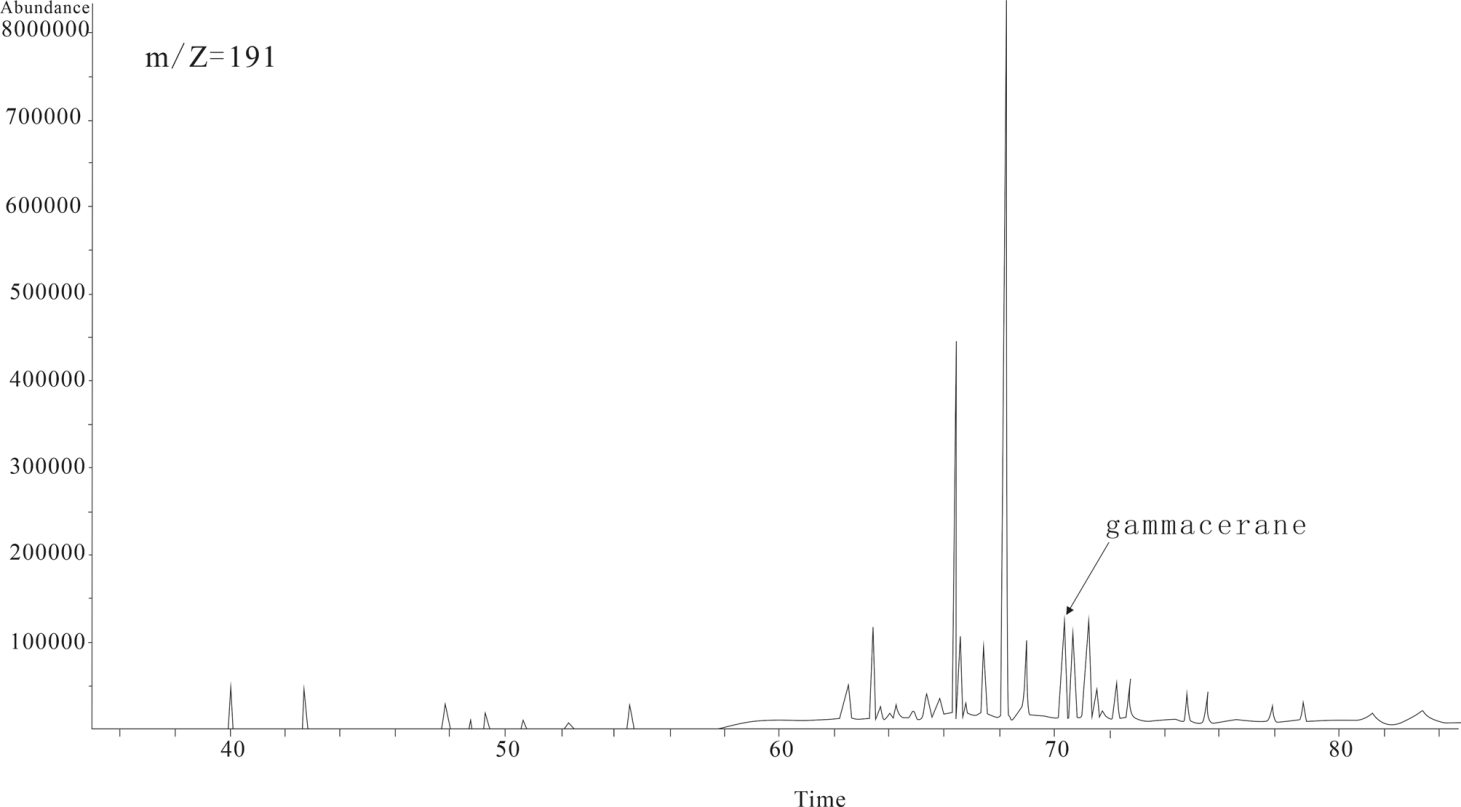
Mass spectrum of m/z = 191.

Ancient water properties, i.e., redox conditions and paleoclimate, play an important role in the formation of oil shale. Therefore, it is necessary to analyze this ancient lake Fig. 5.

#### Analysis of the redox conditions

The redox condition is one of the key factors for organic matter preservation and hydrocarbon generation. A warm, wet and eutrophic water body favors biological productivity, and a reducing water environment favors organic matter preservation and thus the formation of oil shale.

Previous research shows that the values of V/V + Ni and Ni/V can be applied as sensitive indicators of redox conditions in an aqueous medium. Generally, high V/V + Ni and high V/Cr reflect a reducing environment, while low V/V + Ni and low V/Cr reflect a suboxidizing or an oxidizing environment. However, Ni/V decreases as the reducibility of the sedimentary environment increases. Generally, V/V + Ni greater than 0.54 reflects an anaerobic reducing environment, V/V + Ni between 0.46 and 0.54 reflects an oxygen-deficient neutral environment, and V/V + Ni less than 0.46 reflects an aerobic sedimentary environment^13–15^. Generally, Ni/V less than 0.5 reflects a reducing environment, and Ni/V greater than 0.5 reflects an oxidizing environment^14^. In the oil shale samples from the second member of the Bayanerhet Formation, V/V + Ni ranges from 0.64 to 0.81, averaging 0.78, and Ni/V ranges from 0.24 to 0.56, averaging 0.28. These results indicate that the oil shale was formed in a reducing environment.

Some scholars define a Ce anomaly as Ceanom = lg(3Ce N/(2La N + NdN)) and hold that Ceanom greater than 0 suggests Ce enrichment and reflects an oxygen-deficient water body while Ceanom less than 0 suggests Ce depletion and reflects an oxidizing water body. The Ceanom values in oil shale samples are between − 0.02 and 0.009, which suggests that the water body had a weak redox environment.

The REE distribution in sedimentary rocks reflects paleolake conditions of an ancient water body during sedimentation. Particularly, δCe sensitively reveals redox conditions of the sedimentary environment^10,11^. At a certain pH, Ce^3+^ is oxidized to Ce^4+^ in an oxidizing water body, and the Ce^3+^ concentration decreases. Conversely, the Ce^3+^ concentration increases if the water body is oxygen deficient. Therefore, Ce anomalies in a sedimentary system can be used to reflect changes in redox conditions of the water body. Generally, δCe greater than 1 indicates Ce enrichment with a positive anomaly and reflects a reducing environment; δCe less than 0.95 indicates Ce depletion with a negative anomaly and reflects an oxidizing environment. The δCe values of oil shale samples are between 0.97 and 1.04, averaging 1.02, which suggests Ce enrichment (Table 3) and the reducibility of the environment of formation for the oil shale.

#### Paleosalinity analysis

Appropriate salinity potentially promotes the growth and development of microorganisms in a lake and may promote productivity in the lake. Minor elements are most commonly used to infer paleosalinity, with ideal effects achieved.

Sr/Ba is the most common paleosalinity sign. In a freshwater lake, the aqueous medium has high acidity and low mineralization, and Sr and Ba are preserved as bicarbonates in lake water. When the lake water is constantly salinized and mineralization increases, Ba is deposited in the form of barium sulfate first since Sr migrates more easily than Ba does. For Sr, strontium sulfate deposits occur only when lake water or seawater is concentrated to a certain degree^16^. High Sr/Ba indicates high salinity, whereas low Sr/Ba indicates low salinity in a water body^17–19^. The Sr/Ba values of the oil shale samples from the second member of the Bayanerhet Formation average 1.04. Previous research holds that Sr/Ba greater than 1 represents saltwater^20,21^. Therefore, this member was formed in a saltwater environment. During the sedimentation of oil shale, the increase in the salinity of the water body leads to an increase in gammacerane. The existence of a large amount of gammacerane indicates a reducing saltwater environment^22,23^. GC–MS of saturated hydrocarbons revealed gammacerane/αβC30 hopane averages of approximately 0.28, which confirms that the oil shale was formed in a saltwater environment^24^.

#### Lake productivity analysis

Organic matter in lacustrine sedimentary rocks mainly comes from soil organic matter and lake endogenous organic matter, which is far greater than imported soil organic matter. Therefore, it is very necessary to study the lake endogenous productivity for argillaceous source rocks^24–28^. Mo and U are often used to analyze lake endogenous productivity. Generally, high Mo and U abundances represent high productivity, while low abundances represent low productivity^17^. The figure shows an obvious linear relation of Mo and U. Therefore, these parameters could be used to evaluate lake productivity^25^. According to the Mo and U distributions in samples, Mo less than 3.0 and U less than 2.0 reflect low lake productivity, Mo between 3.0 and 4.0 and U between 2.0 and 3.0 reflect medium lake productivity, and Mo greater than 4.0 and U greater than 3.0 reflect high lake productivity. The test results show that the average Mo content is 10.56, and the average U content is 7.54, corresponding to high TOC. These results suggest that lake productivity plays an important role in the enrichment of organic matter^26–29^, but there is no direct linear relation, which means that Mo and U contents as controlling factors are not absolute.

### The ancient lake information history and sequence stratigraphy analysis

The Lower Cretaceous Bayanerhet Formation (K1bt) has parallel bedding, oblique bedding, and associated underwater sliding structures, reflecting characteristics of the shape, occurrence and scale of lacustrine oil bodies intercalated with underwater gravity flow sediments. The scale, thickness, stability, and oil content of oil shale in this region mainly depend on the primitive sedimentary environment of the oil shale i.e., the aforementioned formation conditions of the oil shale^30–32^.

The Lower Cretaceous Bayanerhet Formation (K1bt) in the Bayanjargalan mine area is a complete sequence, among which the first member (K1bt1) was formed in a lowstand period, the second member (K2bt2) was formed in a transgression period and highstand period, and the third member (K3bt3) was formed in a regression period.

(1) Lowstand period: the first member (K1bt1) of Lower Cretaceous Bayanerhet Formation was formed in the lowstand period, the preliminary development period of the basin, and the lithologies are pebbly coarse rocks, sandstone, arenaceous mudstone, and mudstone. This member is dominated by alluvial fan and delta deposits.

(2) Transgression period and highstand period: the second member (K1bt2) of the Lower Cretaceous Bayanerhet Formation was formed during lake level rise caused by constant transgression in a humid climate. The water turned from freshwater to saltwater, lake productivity greatly changed, and abundant organic matter was deposited at relatively high rates, leading to high TOC. Controlled by the first flooding surface (FFS) and the maximum flooding surface (MFS), the sedimentation rate was lower than the accommodation increase rate. According to lithologic and sedimentary characteristics exposed by exploration holes, the lower part of the second member (K1bt2) was formed in a transgression period and is composed of mudstone, arenaceous mudstone, shale, and oil shale locally intercalated with pebbly coarse sandstone, generally fining-upward and showing a typical retrogradational stacking package. The lower part of the second member (K1bt2) has small-scale stratiform-like or lenticular oil shales, which are nonrecoverable and have unstable oil content.

The upper part of the second member (K1bt2) of the Lower Cretaceous Bayanerhet Formation was formed during a highstand period, when the lake water body was still in a relatively humid climate and had high productivity, low salinity, and a moderate sedimentation rate. Given the good preservation conditions, high-quality source rocks were formed, with oil deposits I to V developed. In particular, oil deposits I and II are well developed within the whole basin and occur as stratiform-like oil bodies with good grade and stable distribution. They are recoverable oil shale in this region.

(3) Regression period: third member (K1bt3) of Lower Cretaceous Bayanerhet Formation was formed during water regression period, during which sediments supply rate exceeded lake level rise rate and lake level shrank. Typical progradational sediments were formed from west to east. Sediments become coarser and have more arenaceous component upwards. Thin layers of oil shale are developed at bottom and occur as stratiform-like or lenticular oil bodies with unstable distribution. They are nonrecoverable.

In general, the Lower Cretaceous Bayanerhet Formation (K1bt) in the Bayanjargalan mine area is a complete sequence and was formed during lowstand, transgression, highstand and regression periods. The dominant oil shale deposit was formed in the transgressive systems tract (TST) and the HST, and this deposit has high oil content and stable occurrence.

## Conclusions

The genetic type of the Bayanerhet Formation oil shale in the Bayanjargalan mine area is an inland lacustrine oil shale deposit. Inorganic element analysis and organic geochemical testing are conducted on the oil shale samples collected from three boreholes.

The Bayanerhet Formation oil shale has a relatively high organic content. In boreholes BJ3807, BJ3405 and BJ3005, the average TOC values are 6.53, 7.32 and 8.84, corresponding to oil contents of 5.49%, 6.07% and 7.50%, respectively, indicating oil shale with good quality and development value. Analysis of organic matter sources with biomarkers indicates that lower aquatic organisms such as algae contribute more to the organic matter than higher plants do.

According to research on the values of Fe_2_O_3_/FeO, Rb/Sr and w(La) n/w(Yb)n in the cores from the three boreholes, the Bayanjargalan oil shale is inferred to have formed in a humid paleoclimate with a relatively high sedimentation rate corresponding to high TOC. This result occurs because organic matter is gradually consumed by oxygen in water if the sedimentation rate is low. Consequently, the organic matter content would be low. In contrast, organic matter is quickly sealed by inorganic minerals if the sedimentation rate is high, which favors the preservation and enrichment of organic matter.

In research on the evolution of the paleoaquifer in which the oil shale was formed, the values of Fe^3+^/Fe^2+^, V/V + Ni, Ni/V, Ceanom and δCe are applied as sensitive indicators of the redox conditions in the aqueous medium. These values indicate that the Bayanjargalan oil shale was formed in a water body with a weak redox environment. Moreover, the values of Ca/(Ca + Fe) and Sr/Ba and the values of gammacerane/αβC30 hopane in biomarkers show that the oil shale was formed in a saltwater environment. Analysis of Mo and U shows high endogenous lake productivity, corresponding to high TOC, which suggests that the lacustrine productivity played an important role in the enrichment of organic matter.

In general, the Lower Cretaceous Bayanerhet Formation (K1bt) in the Bayanjargalan mine area encompasses a complete sequence and was formed during lowstand, transgression, highstand and regression periods. The dominant oil shale deposit was formed in the TST and HST, and this deposit has high oil content and stable occurrence. A large set of thick, high-TOC and high-oil-content oil shales in the second member of the Bayanerhet Formation was deposited under such conditions. The abundant terrigenous supply under warm and humid conditions significantly promoted the primitive biological productivity, and the weak redox saltwater environment had a relatively high productivity. All the favorable conditions promoted the formation of high-quality oil shale. The overlying strata were formed when conditions changed and the development of the lake was constrained. Sediments become coarser and have more arenaceous components upward, and the lithology is dominated by a grayish-green argillaceous component and siltstone.
